# MRI assessment of the effects of acetazolamide and external lumbar drainage in idiopathic Normal Pressure Hydrocephalus

**DOI:** 10.1186/s12987-015-0004-z

**Published:** 2015-04-02

**Authors:** Milos Ivkovic, Martin Reiss-Zimmermann, Heather Katzen, Matthias Preuss, Ilhami Kovanlikaya, Linda Heier, Noam Alperin, Karl T Hoffmann, Norman Relkin

**Affiliations:** Weill Cornell Medical College, New York, NY USA; University of Leipzig, Leipzig, Germany; University of Miami School of Medicine, Miami, FL USA

**Keywords:** Normal pressure hydrocephalus, Acetazolamide, External lumbar drainage, MRI, DTI, ASL

## Abstract

**Background:**

The objective was to identify changes in quantitative MRI measures in patients with idiopathic normal pressure hydrocephalus (iNPH) occurring in common after oral acetazolamide (ACZ) and external lumbar drainage (ELD) interventions.

**Methods:**

A total of 25 iNPH patients from two clinical sites underwent serial MRIs and clinical assessments. Eight received ACZ (125-375 mg/day) over 3 months and 12 underwent ELD for up to 72 hours. Five clinically-stable iNPH patients who were scanned serially without interventions served as controls for the MRI component of the study. Subjects were divided into responders and non-responders to the intervention based on gait and cognition assessments made by clinicians blinded to MRI results. The MRI modalities analyzed included T1-weighted images, diffusion tensor Imaging (DTI) and arterial spin labelling (ASL) perfusion studies. Automated threshold techniques were used to define regions of T1 hypo-intensities.

**Results:**

Decreased volume of T1-hypointensities and decreased mean diffusivity (MD) within remaining hypointensities was observed after ACZ and ELD but not in controls. Patients responding positively to these interventions had more extensive decreases in T1-hypointensites than non-responders: ACZ-responders (4,651 ± 2,909 mm^3^), ELD responders (2,338 ± 1,140 mm^3^), ELD non-responders (44 ± 1,188 mm^3^). Changes in DTI MD within T1-hypointensities were greater in ACZ-responders (7.9% ± 2%) and ELD-responders (8.2% ± 3.1%) compared to ELD non-responders (2.1% ± 3%). All the acetazolamide-responders showed increases in whole-brain-average cerebral blood flow (wbCBF) estimated by ASL (18.8% ± 8.7%). The only observed decrease in wbCBF (9.6%) occurred in an acetazolamide-non-responder. A possible association between cerebral atrophy and response was observed, with subjects having the least cortical atrophy (as indicated by a positive z-score on cortical thickness measurements) showing greater clinical improvement after ACZ and ELD.

**Conclusions:**

T1-hypointensity volume and DTI MD measures decreased in the brains of iNPH patients following oral ACZ and ELD. The magnitude of the decrease was greater in treatment responders than non-responders. Despite having different mechanisms of action, both ELD and ACZ may decrease interstitial brain water and increase cerebral blood flow in patients with iNPH. Quantitative MRI measurements appear useful for objectively monitoring response to acetazolamide, ELD and potentially other therapeutic interventions in patients with iNPH.

## Background

Idiopathic Normal Pressure Hydrocephalus (iNPH) is a progressive neurologic disorder in adults in which enlargement of the cerebral ventricles is associated with disturbances of gait, urinary continence and cognition. Standard treatment of iNPH by implantation of a ventricular shunt can arrest progression of symptoms and restore neurologic function in suitably selected patients. Nevertheless, it has been estimated that only 10% to 20% of patients with iNPH receive appropriate specialized treatment [1]. This is due to difficulties in diagnosing iNPH, limits to predicting shunt responsiveness and the morbidity and mortality associated with shunt surgery.

Better diagnostic and prognostic biomarkers are clearly needed to improve clinical management of iNPH. Uncertainties about the underlying disease mechanisms as well as the high prevalence of neurologic comorbidities in iNPH [2] have made the search for such markers very challenging. Adding to the set of challenges, ventriculomegaly and extreme deformations of brain tissue make application of most of the neuroimaging tools that rely on inter-subject image alignment (co-registration) and region-of-interest approaches problematic [3-5]. Further, studying the effects of ventricular shunts by MRI is difficult because shunt valves can be a source of various MRI artifacts.

In this study we evaluated serial MRI scans from patients receiving external lumbar drainage (ELD) or off-label treatment with oral acetazolamide (ACZ), two interventions that do not introduce MR imaging artifacts and are known to produce symptomatic improvements in some iNPH patients. ELD entailed up to three days of controlled removal of CSF via an implanted lumbar catheter and is considered to be among the most definitive prognostic tests for assessing shunt responsiveness [6]. Although the effects of ELD usually abate after a period of several hours to days, longer responses sometimes occur and repeated ELD has been proposed as a long-term treatment alternative to shunt surgery [7]. ACZ is a carbonic anhydrase inhibitor that can be orally administered to decrease CSF production and reduce cerebral interstitial edema. ACZ has been anecdotally reported to alleviate iNPH symptoms [8] and can decrease periventricular white matter T2-hyperintensities in some iNPH patients [9]. ACZ is approved for treating acute mountain sickness, glaucoma and increased intracranial pressure among other diseases, but is not a currently approved treatment for iNPH.

Notable features of this study include: 1.The comparison of two interventions for adult hydrocephalus that act by inherently different mechanisms. 2. The focus on MRI changes associated symptomatic improvement. 3. The use of quantitative MRI analytic methods that are reliable and reproducible. Our goal was to identify reliable MRI markers that can serve as objective indicators of iNPH treatment response and are suitable for future multi-center studies and clinical practice.

## Methods

The data analyzed in this study were obtained in studies approved by the Weill Cornell Medical College (WCMC) IRB and University of Leipzig (UL) Ethical Review Board. Participants gave written informed consent for the analysis of their anonymized MRI scans and associated clinical data. The cohort receiving ACZ is the same as in our previous report [9], but includes additional data from MRI modalities not presented in the previous work. The ELD cohort is part of an ongoing prospective trial underway at University of Leipzig.

### Data acquisition methods

#### ACZ cohort

Selection criteria and dose escalation for the eight iNPH patients who received ACZ was reported previously [9]. In brief, subjects met the International Consensus Criteria for Probable iNPH [10] and were not urgently in need of shunt surgery. Off-label treatment with ACZ started at 125 mg ACZ daily and increased to a maximum of 375 mg over three months. Patients’ demographic data is provided in Table 1. Patients underwent clinical examinations and MRI scans before and after treatment. The outcome was assessed using the Boon Gait Scale [11] (ranging from 2 to 40, higher values indicating greater impairment).

**Table 1:** **Effects of acetazolamide (ACZ) on radiologic and clinical metrics in patients with iNPH**

**ID**	**Age**	**Gender**	**Days between scans**	**Ventricle vol. (mm** ^**3**^ **)**		**Cortical thickness**		**T1 hypo. vol (mm** ^**3**^ **)**		**CBF (ml/100 ml/min)**	**Symmetrized % change**			**Boon score**		**Improvement**
				**Before**	**After**	**mm**	**z-score**	**Before**	**After**	**Before**	**MD**	***f***_***0***_	**CBF**	**Before**	**After**	
**ACZ_1**	90	Male	83	125,124	124,758	2.08	−0.1	13,653	12,087	13.2	9.2	6	35	10	10	yes
**ACZ_2**	72	Female	143	77,447	76,605	2.02	−1.7	19,073	11,782	25.9	8.5	5.7	21	10	6	yes
**ACZ_3**	86	Male	86	123,287	122,220	2.09	−0.2	16,508	13,891	27.2	7	4.5	16	17	12	yes
ACZ_4*	80	Female	99	148,137	146,563	2.29	0.2	78,730	70,205	17.2	6.6	3	22	10	8/15*	yes
**ACZ_5**	76	Male	112	163,105	165,388	2.42	1.7	13,930	13,196	15.8	4.1	2.4	17	12	4	yes
**ACZ_6**	82	Female	93	104,325	105,244	2.20	0.0	7,714	8,525	33.7	−1.6	−1	−9.6	12	14	no
**ACZ_7**	79	Male	136	176,078	176,778	1.99	−1.7	21,785	14,282	16.8	9.6	3.9	19	20	16	yes
**ACZ_8**	79	Female	111	111,200	109,500	2.23	0.1	23,437	19,114	20.6	10.4	9.6	2.9	11	9	yes

*The patient suffered a stroke after the reported MRI scans. Her Boon score initially improved, but then worsened after the stroke.

CBF = cerebral blood flow; *f*_*0*_ = isotropic diffusion fraction [20]; MD = mean diffusivity.

MRI scans were performed on a 3.0 T Signa EXCITE MRI system (General Electric, Waukesha, USA) using an 8-channel head coil: T1-weighted scan by 3D BRAVO sequence (voxel size 1x1x1 mm^3^); DW-MRI with 33 echo-planar diffusion-weighted scans at b = 1000 s/mm^2^ and one at b ≈ 0 s/mm^2^, field of view 230x230 mm, matrix size 128x128, slice thickness 2.5 mm with no gap; ASL sequence with pseudo-continuous labeling [12], post-label delay of 2 s, voxel size 1.8x1.8x3.8 mm^3^. For comparison purposes, data from five iNPH patients that were clinical stable and did not undergo any intervention were included in the analysis. These subjects were scanned serially using the same MRI protocols. Control subject demographic data is provided in the Table 2.

**Table 2:** **iNPH patients scanned serially without interventions (NON = control group)**

**ID**	**Age (at the 1st scan)**	**Gender**	**Days between scans**	**Ventricle vol. (mm** ^**3**^ **)**		**T1 hypo. vol (mm** ^**3**^ **)**		**SPC (%)**
				**Before**	**After**	**Before**	**After**	**MD**
**NON_1**	77	male	512	121,128	124,745	15,813	14,142	3.1
**NON_2**	67	female	181	144,100	147,844	30,720	39,721	−3.8
**NON_3**	71	male	151	93,459	99,813	13,660	19,765	−0.1
**NON_4**	72	female	454	108,616	111,520	10,371	13,991	−1.6
**NON_5**	82	male	552	95,869	99,759	11,251	18,639	0.5

SPC = symmetrized percent change; MD = mean diffusivity.

#### ELD cohort

Twelve consecutive subjects suspected of iNPH underwent ELD for up to 72 hours at the University Hospital in Leipzig, Germany. Patients’ demographic data is given in Table 3. Exclusion criteria were general MRI contraindications, such as patients with pacemaker and metal implants. Before ELD, patients underwent intracranial pressure monitoring for up to 24 hours via an intraparenchymal ICP probe (ICPexpress™, Codman, Johnson & Johnson, MA, USA) in the right frontal lobe which was removed prior to MRI scans. Patients underwent MRI scanning before and after the ELD (time interval between MRI exams was between 4 and 6 days). Scans were performed on a 3.0 T Magnetom Trio (Siemens, Erlangen, Germany) and a 12-channel head coil: T1-weighted scan by MPRAGE sequence (voxel size 1x1x1 mm^3^); DW-MRI protocol had 60 single-shot echo-planar diffusion-weighted volumes at b = 700 s/mm^2^ and 10 scans at b ≈ 0 s/mm^2^; field of view 256x256 mm; matrix size 128x128; slice thickness 2 mm. ASL imaging was not performed on the ELD cohort. The binary (yes/no) assessment of response after ELD (Table 3) was based on clinician assessment incorporating patient/caregiver feedback, results of Mini Mental Status Exam [13] and a standardized 10-meter gait test (number of seconds it takes to walk 10 m) derived from the Boon scale [14]. The assessments were made blinded to the results of quantitative MRI analyses.

**Table 3:** **Effects of extended lumbar drainage (ELD) on radiologic and clinical metrics in patients with iNPH**

**ID**	**Age**	**Gender**	**CSF drainage (ml)**	**Ventricle vol. (mm** ^**3**^ **)**		**Cortical thickness**		**T1-hypo. vol. (mm** ^**3**^ **)**		**Symmetrized % Change**		**MMSE**		**10 m gait test (secs)**		**Mean ICP**	**Rout CSF**	**Improvement**
				**Before**	**After**	**mm**	**z-score**	**Before**	**After**	**MD**	***f***_***0***_	**Before**	**After**	**Before**	**After**	**(mmHg)**	**(mmHg/ml/min)**	
**ELD_1**	70	male	410	282,634	252,228	2.38	1.3	21,772	18,966	8.3	5.2	26	20	na	na	11.2	14.7	Yes
**ELD_2**	76	male	515	222,300	189,744	2.15	0.3	37,581	34,730	7.1	5.3	26	27	na	29	11.7	22.8	Yes
**ELD_3**	75	male	640	89,100	54,547	2.30	1.0	9,921	5,896	14.1	6.4	29	25	24	23	5.3	3.4	Yes
**ELD_4**	65	male	516	165,969	159,925	2.21	−1.1	27,415	25,348	4.2	3.2	30	30	nm	nm	5.3	nm	Yes
**ELD_5**	74	female	350	129,471	120,182	2.38	1.5	17,427	15,244	6.4	0.8	17	20	22	13	9	21.6	Yes
**ELD_6**	76	male	487	112,852	106,816	2.20	0.9	6,692	6,732	6.0	3.1	30	30	14	13	8.5	14.9	Yes
**ELD_7**	73	female	583	162,758	155,040	2.37	1.4	18,451	15,975	11.2	4.2	30	29	15	14	9.8	12.8	Yes
**ELD_8**	70	male	398	103,008	104,376	1.85	−2.3	19,693	18,061	1.3	0.4	21	16	20	20	1.6	nm	No
**ELD_9**	76	female	566	73,976	74,968	2.17	−0.2	7,546	6,805	6.8	6.6	24	29	na	na	2.9	9.6	No
**ELD_10**	73	female	260	54,910	55,503	2.20	0.1	4,869	4,407	−2.4	−0.8	29	29	11	20	3.8	7.8	No
**ELD_11**	70	female	262	104,398	98,346	2.21	0.1	29,650	30,622	3.5	1.8	28	29	24	27	6.1	7.5	No
**ELD_12**	83	male	344	143,898	135,933	2.18	0.1	14,253	15,896	1.5	0.2	29	30	13	12	9.8	14.2	No

MD = mean diffusivity; *f*_*0*_ = isotropic diffusion fraction [20]; MMSE = Mini Mental Status Exam; ICP = intracranial pressure; Rout CSF = resistance to CSF drainage; na = not able to perform test; nm = not measured.

### Analysis methods

#### Volumetric analysis

Brain tissue segmentation with automatically detected T1-hypointensities (white matter abnormalities) and cortical surface reconstruction was performed by FreeSurfer software suite [15]. Manual corrections of the FreeSurfer segmentations were performed by a neuro-radiologist (I.K. 20 years of experience) where needed. After the segmentation of the individual scans, automated single-subject longitudinal processing [16] was performed on the scans before/after intervention. Total ventricle volume was obtained by summing volumes of the relevant FreeSurfer’s segmentation regions.

Patients’ cortical thickness was compared to gender and age matched healthy control subjects (patient age ± 5 years) from the Alzheimer’s Disease Neuroimaging Initiative (ADNI) repository. The analysis employed a general linear model implemented in the FreeSurfer’s QDEC (Query, Design, Estimate, Contrast) application. Positive z-scores represent cortical thickness higher than average, negative z-scores indicate above-average atrophy.

#### Diffusion-weighted MRI

Eddy current and motion correction were performed by aligning of the gradient volumes to low gradient (“b_0_”) volume [17] followed by a corresponding adjustment of the diffusion gradient vectors [18]. Comparison of the DW-MRI data before/after intervention was done by aligning the b_0_ images (by a diffeomorphic method [19]). To analyze changes within white matter classified as pathologic by FreeSurfer (T1-hypointensities), we aligned the T1 and b_0_ scans (of the same patient, at the same time point). We deliberately avoided inter-patient image alignment, which can be problematic in general [3] and particularly in iNPH because of variable degree of ventriculomegaly and pertinent large tissue deformations [4,5].

In addition to the standard DTI indices, we estimated differences in DW-MRI data by the ball-and-stick model [20], a popular variant of the multi-tensor model that estimates signal contribution from the isotropic diffuison component, associated with unbound extracellular water (denoted *f*_*0*_), and the signal fractions associated with axonal bundles. This model was applied because pervientricular white mater surrounding the frontal and occipital ventricle horns (subject to pathologic water accumulation in iNPH) can have multiple axonal orientations within a voxel. We sought to investigate if presence of multiple axonal bundles could affect the obtained DW-MRI statistics. The ball-and-stick model with up to two automatically detected axonal bundles yields the highest number of parameters that can be reliably estimated with the available single b-shell, 33 directions DW-MRI data [20].

#### Cerebral blood flow

The cerebral blood flow (CBF) maps for the ACZ subjects only were derived from the ASL scans [21] and aligned to the T1-weighed images. FreeSurfer’s tissue segmentation was used to mask brain parenchyma on the CBF maps. Cerebellum was excluded from the subsequent analysis because its coverage by the pulsed-continuous method is variable and subject to error [22]. Global differences in brain parenchyma CBF before/after intervention are reported in terms of Symmetrized Percent Change (SPC) [16]. In order to associate positive numbers with presumed positive outcome (increased blood perfusion after intervention); SPC for the CBF was defined by subtracting values before an intervention from the values after the intervention. On the other hand, SPC for the DW-MRI metrics were calculated by subtracting values after the intervention from the values before the intervention (so that positive SPC on MD means that mean diffusivity decreased after the intervention).

## Results

### Change in clinical scores

Among patients receiving ACZ: six out of eight patients experienced improvement in clinical symptoms (Table 1). The oldest patient (90 years) did not demonstrate observable change in clinical symptoms. Another patient who did not improve (ACZ_6) was in a hypertensive crisis at the time of the follow-up visit (220/100 mmHg) and acknowledged having discontinued prescribed anti-hypertensive treatment weeks before. Another patient suffered a stroke while on the ACZ treatment, but this occurred after the Boon Gait testing and a follow-up MRI exam reported in Table 1. Her Boon score initially improved on ACZ, then worsened after the stroke (Table 1). In the previous work [9] we reported metrics derived from an MRI study done after the stroke, in this paper we use Boon score and an MRI study done while she was receiving ACZ and before the stroke (the follow-up study was done 99 days after the baseline and 82 days after the patient started ACZ treatment).

The ELD cohort: seven patients improved by a subjective assessment and five patients did not (Table 3). Only two patients improved MMSE by more than one point. Three patients were not able to perform the gait test before and after ELD. One patient was able to perform gait test after ELD, but not before.

### MRI volumetric changes

**Figure 1:**
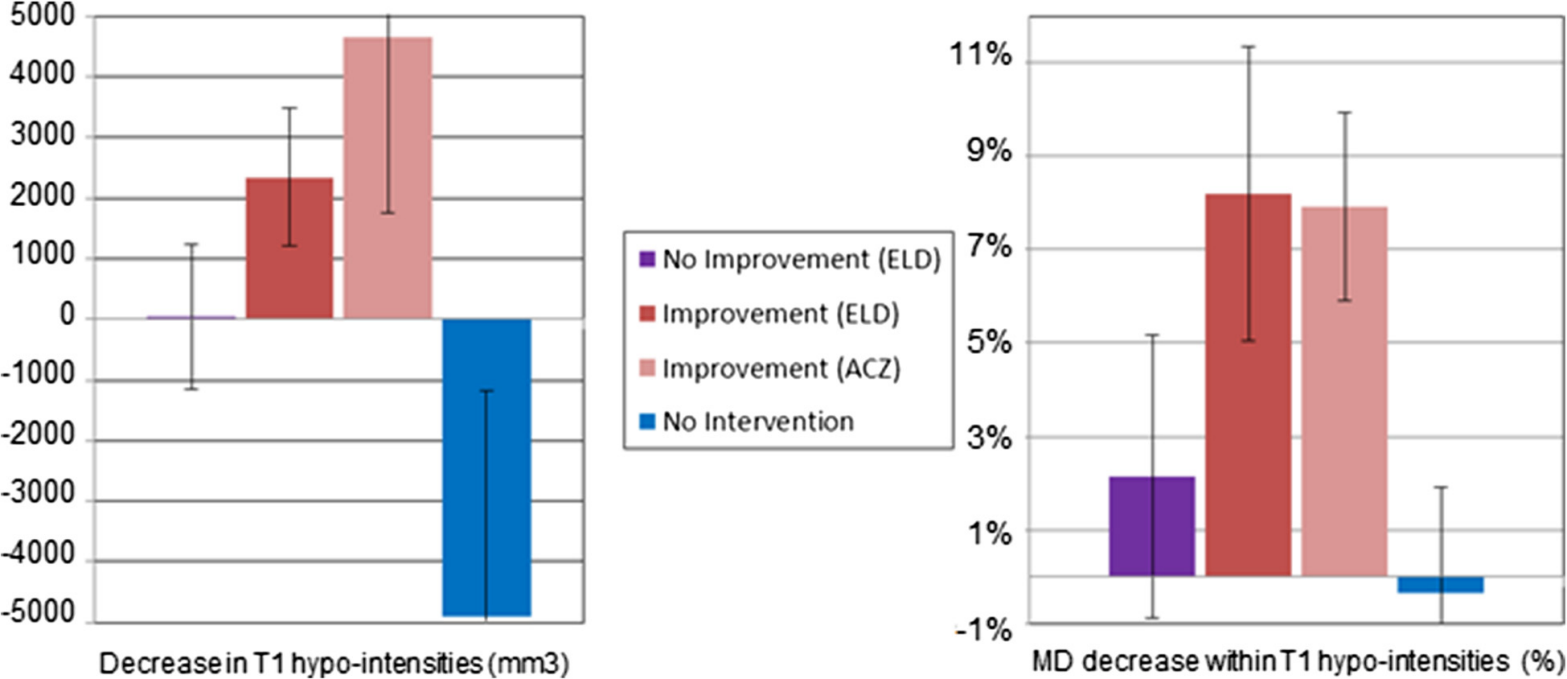
**Observed changes in T1-hypointensities and mean diffusivity after treatment. Left:** Decrease in volume of T1-hypointensities (y axis) was higher among patients that experienced clinical improvement (red bar, ELD-improvement (N = 7) vs. ELD-no-improvement (purple bar set to zero N = 5): *p* = 0.008, and between ACZ-improvement (pink bar, N = 7) relative to no-intervention (blue bar, N = 5): *p* = 0.002). Patients that were not subject to intervention showed an increase in T1-hypointensities (blue bar). **Right:** Percentage decrease in DW-MRI mean diffusivity (MD) within remaining T1-hypointensites was higher in patients that experienced clinical improvement (ELD-improvement vs. ELD-No-Improvement: *p* = 0.007, ACZ vs. No-Intervention: *p* = 0.0002). Data are means +/− SEM.

In both ACZ and ELD cohorts, most subjects showed decreased volume of T1-hypointensities after treatment. The extent of decrease was quantitatively greater in subjects with symptomatic improvement than in non-responders: one-sided t-test comparing ELD responders and non-responders *p* = 0.008. In contrast, four out of five control patients showed increases in hypo-intensities after the second scan (Table 2). This was significantly different from the seven ACZ-responders, one-sided t-test *p* = 0.002 (Tables 1 and 2, Figure 1).

There was one patient who responded positively to ELD, but did not have a decrease in T1-hypointensity (ELD_6). This patient had the smallest volume of baseline T1-hypointinsities among the responders and had a maximal score (no impairment) on MMSE before and after ELD. The ACZ-treated patient who eventually suffered a stroke had the highest volume of T1-hypointensities among all the patients in both cohorts. An ACZ-treated patient with acute hypertension at the time of the follow-up scan (ACZ_6) had increased volume of T1-hypointensities compared to the baseline (Table 1). As previously reported [9], ventricular volume did not change significantly in ACZ-treated subjects. It is noteworthy that the patient with the highest volume of drained CSF also had the greatest reduction in ventricle size (patient ELD_3 in Table 3), but there was no clear association between ventricle volume change and the amount of drained CSF or symptomatic response. This is consistent with previously reports that change in ventricular volume does not correlate with outcome of iNPH treatment [23] or the amount of drained CSF [24,25].

A possible association between cerebral atrophy and response emerged: Patients with the least cortical atrophy (indicated by a positive z-score on cortical thickness) were the best responders in terms of clinical improvement. This observation applies to both the ACZ and ELD cohorts (Tables 1 and 3). Further, average cortical thickness z-score for ELD responders was 0.24, versus −0.1 for non-responders. Changes in the whole-brain average cortical thickness before/after treatment were within rounding error.

### Diffusion-weighted-MRI changes

Post-intervention changes in axial diffusivity, radial diffusivity, mean diffusivity (MD) and fractional anisotropy, averaged over the whole-brain, were less than 2% and subject to change depending on the parameters of alignment, motion correction and skull stripping algorithms. However, the decrease in MD and isotropic water fraction (*f*_*0*_ in the ball-and-stick model) within the remaining T1-hypointensities was higher than in the brain parenchyma overall and statistically significantly higher in ELD-responders compared to non-responders (difference in mean diffusivity symmetrized percent decrease one-sided t-test, *p* = 0.007, data in Table 3) and even higher in ACZ-responders compared to the patients that did not undergo intervention, one-sided t-test *p* = 0.0002 (data in Tables 1 and 2). Of note, the patient with acute hypertension (ACZ_6) did not exhibit decrease in MD. An elevation in venous pressure may explain decreased CSF absorption [26].

**Figure 2:**
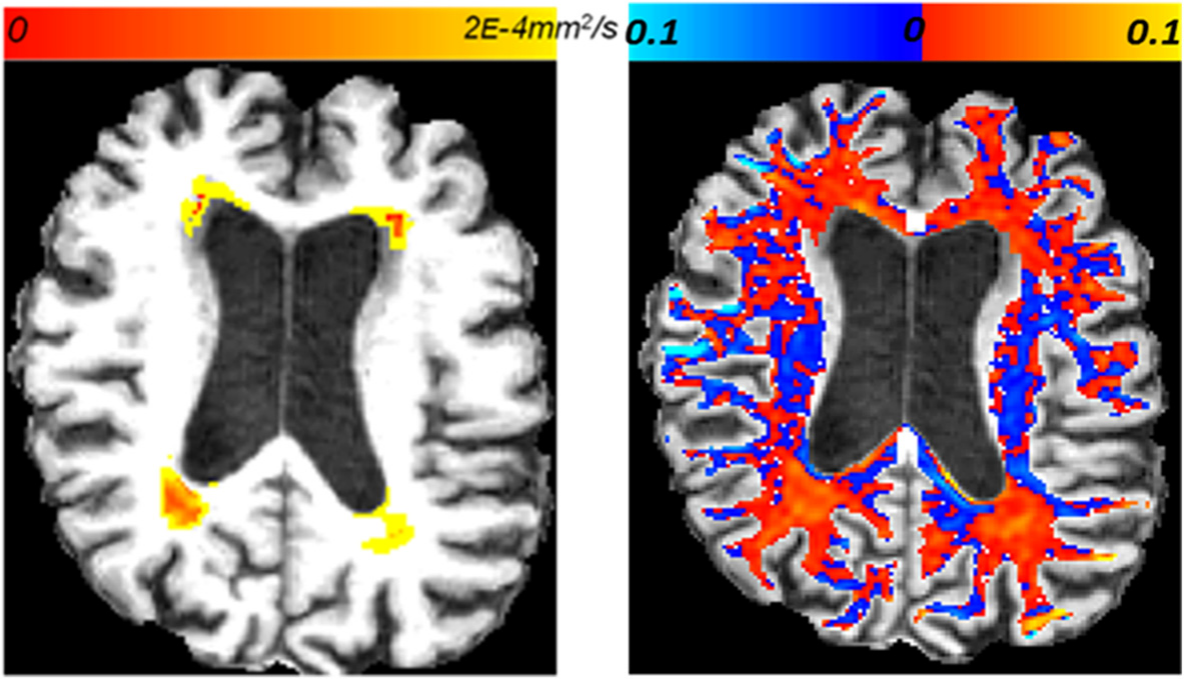
**Illustration of DW-MRI changes location. Left:** Regional changes in mean diffusivity within T1-hypointensities, before *versus* after the acetazolamide treatment for patient ACZ_3. **Right:** Changes in isotropic water fraction, within white matter, on the same brain slice. Red indicates decrease in the isotropic water fraction, blue indicates increase.

**Figure 3:**
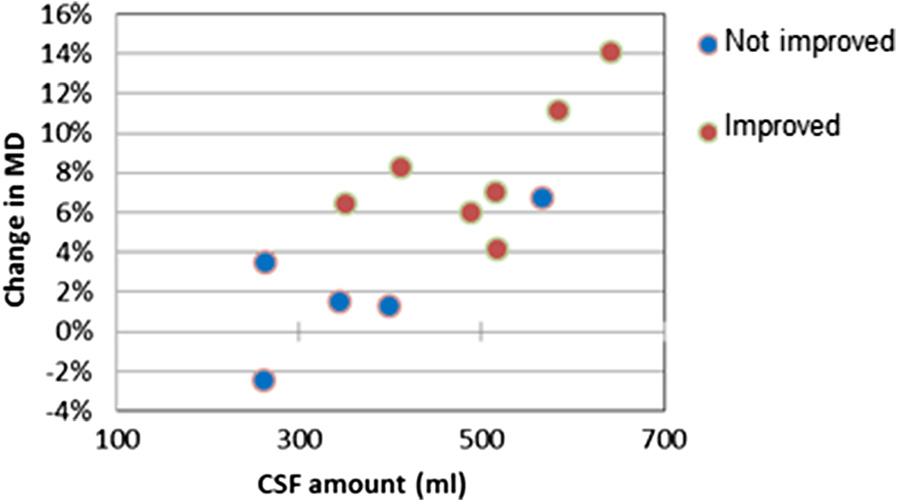
**Plot of symmetrized percent change in mean diffusivity (MD) within the remaining T1-hypointensities against volume of CSF drained in patients treated with external lumbar drainage.** The outlier among the non-responding patients (by having high volume of drained CSF and high decrease in MD) is ELD_9. Her gait problems were attributed to Parkinson’s disease.

We also noticed slight increase in MD and isotropic water fraction within the internal capsule (blue regions on Figure 2) of the responders. This is analogous to the well-documented increase in radial diffusivity in parts of the internal capsule after shunt surgery (see [5] and references therein) and after ELD (our findings, with further references, are published in [27]). It has been shown numerically that the concave shape of the ventricles can create compressive stress in the brain parenchyma of untreated iNPH patients [28], which could be relieved by treatment. There was an intriguing correlation between MD change within post-intervention T1-hypointensities and the volume of the CSF drained during ELD (Figure 3), but further study and more patients are needed to clarify the relationship between ELD and change in interstitial water. We expect that the time-interval between the end of ELD and the follow-up MRI scan is an important covariate, similarly to volumetric changes following CSF removal [25].

### Cerebral blood flow changes

**Figure 4:**
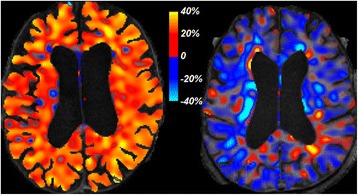
**Symmetrized percent change in cerebral blood flow (CBF) after acetazolamide treatment. Left:** a patient with normal blood pressure (ACZ_1). **Right:** the patient with acute hypertension (ACZ_6) did not experience increase in CBF.

All the ACZ patients, except for the patient with hypertension, exhibited a global increase in CBF (Table 1 and Figure 4, suggesting that improved blood perfusion lead to symptomatic improvements resulting from ACZ. Our observation for the patient with hypertension is consistent with the observation that iNPH patients who do not respond favorably to shunt placement tend to have higher frequency of hypertension [29]. The average global CBF value for our iNPH patients (21.3 ± 6.6 ml/100 ml/min) was lower than previously reported CBF estimates obtained by PET, 36.5 ± 10.5 ml/100 ml/min in [30]. This could be due to age differences (our cohort mean age 81 ± 5.4 years, versus 67 ± 11 years in [30]), but an answer would require accounting for patient-specific labeled bolus arrival time to target tissue (ATT), since ATT varies greatly among elderly and even between vascular territories within the brain of a single subject [31,32]. The ASL protocol employed had a single post-label delay, so it was not possible to estimate ATT jointly with CBF. Because of this, we have reported CBF changes for each subject individually, without inter-subject comparison.

## Discussion

**Figure 5:**
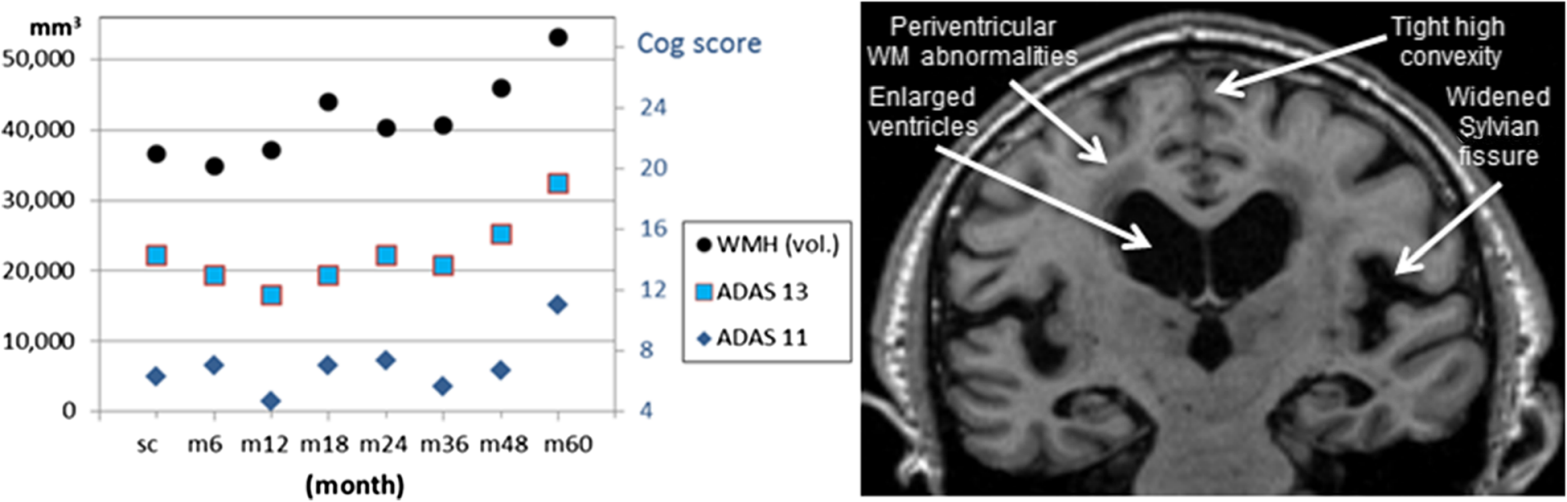
**A subject with remarkable long-term correlation between cognitive scores and T1-hypointensities volume. Left:** Correlation between Alzheimer’s Disease Assessment Scale (ADAS) cognitive scores and the volume of the white matter T1-hypointensities, over a 5 year period, for the Alzheimer’s Disease Neuroimaging Initiative (ADNI) subject 0644 (an MCI case). All the data points were independently calculated and available at the ADNI portal [39]. **Right**: Hallmarks of CSF dysfunction on the same subject: ventriculomegaly and DESH pattern. Note lacunae in the deep gray matter, consistent with the radiologic presentation of dilated veins observed in untreated NPH patients [40].

Among the MR imaging modalities included in this study, arguably the most informative is T1. In addition to well-known iNPH markers, Evans’ index and recently validated DESH pattern [33] it offers the possibility to perform automatic volume estimates validated across MRI-platforms on older subjects [34]. These volume estimates can be compared to (nowadays widely available) control subjects for a quantitative estimate of the level of brain atrophy. Atrophy estimates may contribute to the prediction of shunt-responsiveness. Further, automatic labeling of white matter T1-hypointenisities showed sensitivity to changes in iNPH-related white matter pathology, similar to FLAIR sequence [9]. It is interesting to mention that a long-term connection between the withdrawal of white matter lesions and improvement in clinical symptoms can be observed in some patients: Figure 5 shows the correlation between the Alzheimer’s Disease Assessment Scale-cognitive subscale (ADAS-cog) [35] and the volume of T1 white matter hypo-intensities for the ADNI subject 0644. This patient has DESH pattern (Figure 5) and we hypothesize that some of the variability in clinical symptoms (non-monotonic decline) seen in certain AD patients [36] is related to CSF (and vascular) dysfunction and rather than primary AD pathology. One caveat in the interpretation of our findings is that every currently available definition of T1-hypointensities (or FLAIR hyperintensities) is essentially an *ad-hoc* convention. We used FreeSurfer’s definition because this post-processing suite is well-known and widely used.

By using DW-MRI, it was possible to detect a decrease in mean diffusivity in the white matter regions that remained classified as pathologic after intervention. This indicates that those regions were affected by the interventions and may even experience greater decreases in abnormal accumulation of water compared to the brain parenchyma overall. Note that this information cannot be obtained from T1 or FLAIR images. It must be noted, however, that while the observed numerical changes in DW-MRI statistics indicate trends, they do not provide quantitative information in terms of the interstitial water volume decrease. We did not see advantages to using isotropic volume fraction compared to MD, or *vice-versa*.

CBF in iNPH patients has been studied by DSC-MRI, PET and SPECT [24,30,37], but the first publication on iNPH using ASL appeared only very recently [38]. This was a study of CBF before and after CSF tap test that employed an ASL protocol with shorter post-label delay than ours (1.6 s vs. 2 s in our study). ASL’s big advantage, compared to DSC-MRI and tomography perfusion techniques, is that it does not require artificial contrast and is non-invasive; however, this advantage comes with an important methodological shortcoming*:* ASL CBF estimates suffer from low signal-to-noise ratio, especially in the white matter [31]. Further, non-conclusive results in [38] reinforced our opinion that an ASL protocol with multiple post-label delays, capable of resolving both CBF and ATT, seems necessary for regional CBF estimates and inter-subject comparison among iNPH patients.

### Further methodological shortcomings

The small number of subjects is a shortcoming of this study. Patient cohort size is an issue in many iNPH studies, due in part to the relatively low rate of diagnosis of this disease [1]. Another issue that can affect the interpretation and generalizability of the present results is that although the ACZ and ELD treated iNPH cohorts were identified using common diagnostic criteria, assignment to the respective treatment arms depended on the center at which subjects were evaluated, creating a potential selection bias. The clinical outcome measures at the two centers were similar, but not identical. These issues should be addressed in future studies that enroll larger numbers of iNPH subjects and employ enhanced MRI protocols.

## Conclusion

Small cohorts and inconsistent assessment protocols are problems hampering NPH research. Yet, despite different medical centers, different MRI protocols and very different interventions, we observed very similar MRI changes linked to symptomatic improvement. T1-weighted MRI scans are useful in multiple ways: In addition to atrophy estimates, automatically labeled T1-hypointenisities showed sensitivity to changes in iNPH-related white matter pathology. DTI offers possibility to detect changes in water accumulation within T1-hyperintensities, information that is not obtainable by T1 (or FLAIR) protocols. ASL MRI could help explain physiologic origins of symptoms withdrawal, but protocols with multiple wait-delays seem necessary.

## Abbreviations

ACZ: Acetazolamide
ADAS-cog: Alzheimer’s disease assessment Scale-cognitive subscale
ADNI: Alzheimer’s disease neuroimaging Initiative
ASL: Arterial spin labelling
ATT: Arrival time to target tissue
CBF: Cerebral blood flow
DTI: Diffusion tensor imaging
DW-MRI: Diffusion weighted MRI
DSC-MRI: Dynamic susceptibility contrast MRI
ELD: External lumbar drainage
FLAIR: Fluid attenuated inversion recovery
iNPH: Idiopathic normal pressure hydrocephalus
ICP: Intracranial pressure
MD: Mean diffusivity
MMSE: Mini mental status exam
SPC: Symmetrized percent change
